# Response to Braillon

**DOI:** 10.1093/jncics/pkab005

**Published:** 2021-01-23

**Authors:** Bernardo Bonanni, Davide Serrano, Patrick Maisonneuve, Matteo Lazzeroni, Powel H Brown, Aliana Guerrieri-Gonzaga, Eva Szabo

**Affiliations:** 1 Division of Cancer Prevention and Genetics, IEO European Institute of Oncology IRCCS, Milan, Italy; 2 Division of Epidemiology and Biostatistics, IEO European Institute of Oncology IRCCS, Milan, Italy; 3 Department of Clinical Cancer Prevention, The University of Texas MD Anderson Cancer Center, Houston, TX, USA; 4 Division of Cancer Prevention, National Cancer Institute, Bethesda, MD, USA

We thank Dr Braillon for his comments on our recent phase IIb trial on the efficacy of low-dose aspirin to reduce the size of subsolid lung nodules. Aspirin has a long history of pros and cons regarding its role in cancer prevention, primarily for colorectal cancer but potentially also for cancers arising in other organs. At the time of the planning and performance of our study, the recent publication by Rothwell et al. (1) suggested the protective role of aspirin during early lung carcinogenesis. The rationale for our trial was that if the Rothwell meta-analyses showed divergence in lung cancer mortality within 5 years of aspirin use, then the effect on premalignancy might be assessable within 1 year of usage. Preclinical studies similarly supported the testing of aspirin in a high-risk population (2). The more recent results of the ASPirin in Reducing Events in the Elderly (ASPREE) trial suggest that aspirin may, instead, increase mortality in an elderly population, but these data were not available at the time we conceptualized our trial, and the results of ASPREE were clearly unexpected. There also remains the possibility that aspirin has different effects during the early and late stages of carcinogenesis, with the ASPREE trial being heavily weighted toward late carcinogenesis, whereas trials in younger populations are more likely to address the earlier phases (3). The data on the age of our population were included in Supplementary Table 1 (available online). Only 7 (14.3%) and 5 (10.2%) participants older than age 70 years were treated with placebo and aspirin, respectively. This was the age group for non-Black and non-Hispanic participants in the ASPREE trial. Certainly, age has to be taken into account for aspirin in prevention as suggested by Cuzick at al. (4).

We agree with Dr Braillon that primary prevention should involve multiple aspects starting from avoiding environmental exposures, to lifestyle, and possibly, medical interventions. The COSMOS trial, which was the main source of accrual for our study, required yearly smoking cessation counseling by a trained psychologist for all participants. In our trial, 8 (12.1%) of the 66 current smokers quit within the year of treatment, but 3 (6.3%) of the 32 former smokers restarted. Although other studies have focused on smoking cessation, showing variable success in quitting smoking with a variety of interventions, we chose to focus on a logical pharmacologic intervention as the main experimental variable. It should be noted, however, that the benefits of smoking cessation on lung cancer mortality take a long time to be realized even in those who do manage to successfully quit smoking. In the Lung Health Study, only the long-term follow-up after 14.5 years showed a decrease in lung cancer mortality after sustained quitting; a shorter follow-up of 5 years did not show any effect of quitting on lung cancer mortality (5,6). The effect of smoking cessation on lung premalignancy is even less documented and remains to be determined.

## Funding

The study object of this letter was funded by NCI Contract No. HHSN261201200034I

## Notes

**Role of the funder:** The funder had role in the study design, data interpretation, and manuscript writing. The funder had no role in the collection or analysis. The decision to submit the letter has been shared between the principal investigator and the funder.

**Disclosures:** The authors have no conflicts of interest to disclose.

**Author contributions:** All—study design letter writing, revision

## Data Availability

The data underlying this letter will be shared on reasonable request to the National Cancer Institute at the following website: https://cdas.cancer.gov/learn/eppt/browse/.
